# Personal exposure to particulate matter and heart rate variability among informal electronic waste workers at Agbogbloshie: a longitudinal study

**DOI:** 10.1186/s12889-021-12241-2

**Published:** 2021-11-25

**Authors:** Afua A. Amoabeng Nti, Thomas G. Robins, John Arko Mensah, Duah Dwomoh, Lawrencia Kwarteng, Sylvia A. Takyi, Augustine Acquah, Niladri Basu, Stuart Batterman, Julius N. Fobil

**Affiliations:** 1 Department of Biological, Environmental & Occupational Health Sciences, School of Public Health, University of Ghana, P.O. Box LG13, Accra, Ghana; 2 Department of Environmental Health Sciences, University of Michigan, 1415 Washington Heights, Ann Arbor, MI 48109 USA; 3 Department of Biostatistics, School of Public Health, University of Ghana, P.O. Box LG13, Accra, Ghana; 4 Faculty of Agricultural and Environmental Sciences, McGill University, Montréal, QC H9X 3V9 Canada

**Keywords:** Personal particulate matter, Informal e-waste recycling, PM_2.5_, PM_10_, PM _10–2.5_, Cardiac function, Heart rate variability, Agbogbloshie, Madina-Zongo

## Abstract

**Background:**

Informal electronic waste recycling activities are major contributors to ambient air pollution, yet studies assessing the effects or relationship between direct/continuous exposure of informal e-waste workers to particulate matter and cardiovascular function are rare.

**Methods:**

Repeated measurements of fractions of PM_2.5_, PM_10–2.5_, and PM_10_ in personal air of informal e-waste workers, (*n* = 142) and a comparable group (*n* = 65) were taken over a period of 20 months (March 2017 to November, 2018). Concurrently, 5-min resting electrocardiogram was performed on each participant to assess resting heart rate variability indices. Linear mixed-effects models were used to assess the association between PM fractions and cardiac function.

**Results:**

SDNN, RMSSD, LF, HF and LH/HF ratio were all associated with PM. Significant associations were observed for PM_2.5_ and Mean NN (*p* = 0.039), PM10 and SDNN (*p* = 0.035) and PM 10–2.5 and LH/HF (*p* = 0.039). A 10 μg/m^3^ increase in the concentrations of PM _2.5_, PM_10–2.5_, and PM_10_ in personal air was associated with reduced HRV indices and increased resting HR. A 10 μg/m^3^ per interquartile (IQR) increase in PM_10–2.5_ and PM_10_, decreased SDNN by 11% [(95% CI: − 0.002- 0.000); (*p* = 0.187)] and 34% [(95% CI: − 0.002-0.001); (*p* = 0.035)] respectively. However, PM_2.5_ increased SDNN by 34% (95% CI: − 1.32-0.64); (*p* = 0.493). Also, 10 μg/m^3^ increase in PM_2.5_, PM_10–2.5_ and PM_10_ decreased RMSSD by 27% [(− 1.34–0.79); (*p* = 0.620)], 11% [(− 1.73, 0.95); (*p* = 0.846)] and 0.57% [(− 1.56–0.46); (*p* = 0.255%)].

**Conclusion:**

Informal e-waste workers are at increased risk of developing cardiovascular disease from cardiac autonomic dysfunction as seen in reduced HRV and increased heart rate.

**Supplementary Information:**

The online version contains supplementary material available at 10.1186/s12889-021-12241-2.

## Introduction

Increasing evidence implicates informal electronic waste (e-waste) recycling activities as a major contributor to ambient air pollution and yet studies describing the effects of continuous exposure to airborne particulates on cardiovascular (CV) function of informal e-waste workers are unavailable. Studies have associated air pollution with adverse CV outcomes such as increased heart rate and reduced heart rate variability (HRV) [1–3], ventricular tachycardia [4], vascular tone [5, 6] and morbidity and mortality due to cardiac arrhythmias [7, 8].

Reduced heart rate variability (HRV) is reported as an independent predictor of CV disease and death across diverse populations [9–11]. It is a marker for poor health and increased risk for cardiac events [12]. Reduced HRV is a prognostic factor of increased risk for all-cause and CV mortality [13–15]. Previous studies have related reduced HRV with CV outcomes such as myocardial infarction [3, 16, 17] and coronary artery disease [18]. Heart rate or rhythm abnormalities without hypoxia or respiratory distress in response to exposure to airborne particulates have been observed in many studies [19–21]. Other studies have consistently shown that inhaled airborne PM_2.5_ and PM_10_ are associated with lower HRV in infants, the elderly, individuals with compromised CV integrity, as well as healthy individuals [12, 22–26]. Numerous studies have examined HRV in association with the total mass of PM_2.5_ and PM_10_ in various populations. A meta-analysis estimated the pooled effects of the total mass of PM_2.5_, finding a 1–2% decrease in HRV associated with each 10 μg/m^3^ increase in PM_2.5_ concentration [24]. However, associations between PM_2.5_ and cardiovascular-related markers have been reported by only a few studies [27, 28]. Few occupational studies have also established association between reduced HRV and exposure to airborne PM_2.5_ and PM_10_ [29–31].

E-waste recycling at Agbogbloshie in Accra, Ghana is conducted in an unhealthy and unsafe environment. The Blacksmith Institute, now Pure Earth has classified the site among the 10 most polluted areas on earth [32]. Due to the geographic layout, prevailing wind conditions, and intensity of smoke production due to open air burning of e-waste, there is a great public health concern regards to health implications of these exposures among e-waste workers and nearby populations. Open-air burning of cables and manual dismantling of lead-acid batteries are common practices, and these activities could result in significant environmental pollution and impact negatively on human health. Due to the improper processing of e-waste, workers are commonly exposed to toxic chemicals and are at increased risk of several health effects. Previous studies have documented evidence of several pollutants in the soil [33], water [34], and biological materials (blood, urine, breast milk) of both workers and by-standers [35–37]. Recent air pollution data has reported very high concentrations of PM in ambient air [38, 39] and personal air of the waste recyclers [40–42]. The levels were above the WHO-24 h air quality standards of 25 μg/m^3^ and 50 μg/m^3^ for PM_2.5_ and PM_10_ as well as the background levels of 30 μg/m^3^ for PM_2.5_ in the city of Accra. Informal e-waste workers at Agbogbloshie may be at higher risk of developing PM-related cardiovascular diseases or experiencing premature deaths due to inflammation-induced cardiac autonomic dysfunction. The link between inhalation of ambient air particles and effects on cardiac rhythm may be the induction of an inflammatory response in the lung with a subsequent release of chemical mediators that alter the autonomic nervous system’s control of cardiac rhythm [43]. Our study aimed to investigate the association between direct exposure to elevated levels of ambient particulates in the breathing zone of informal e-waste workers and alterations in cardiac autonomic function control. Specifically, this study was designed to determine whether high concentrations of PM is associated with the impaired cardiovascular function, as measured by the analysis of heart rate (HR), HRV and blood pressure.

## Materials and methods

### Study design and study area

A longitudinal panel study design with repeated data collection at four sampling points for personal PM and health outcomes among e-waste workers at Agbogbloshie e-waste and comparable population was selected from populations at Madina Zongo was employed for this study. By the Ghana Statistical Service’s definition [44], both Agbogbloshie and Madina Zongo are classified as slum communities. Round I of data collection was done from (March–May 2017), round II (August–October 2017), round III (January–April 2018) and round IV from (August–November 2018).

The Agbogbloshie e-waste recycling site is located in the central business district of Accra, Ghana’s capital, surrounded by commercial centers, densely populated residential areas, the Odaw river and Korle lagoon. The Agbobgloshie electronic scrap yard, however, has become famous for its pollution and contamination of the environment, especially due to informal e-waste recycling activities. E-waste recycling processes include manual dismantling and open-air burning of e-waste fragments, enhanced by fire accelerants such as Styrofoam and tires to retrieve metals such as copper, aluminum, and gold for sale [32, 45, 46]. This continuous and unrestricted burning, however, produces plumes of black toxic smoke, containing several mixtures of hazardous substances such as PM, into the atmosphere [45]. Informal e-waste workers and the Agbogbloshie community in general are at increased risk of several health conditions including e-waste related cardiovascular health effects from the continuous inhalation of airborne particulates present in the atmosphere.

The comparable or e-waste unexposed site, Madina Zongo, is also a densely populated suburb located at the La Nkwantanang Municipality; more than 15 km from the Agbogbloshie e-waste site. Majority of the residents of Madina Zongo, like Agbogbloshie, are migrants from northern Ghana, and are Moslems. The community members are mainly traders, engaged in a diverse mix of small-scale, informal businesses, mostly petty trading of items such as used clothing, charcoal, cold drinks, street foods, bicycle spare parts, staple foods, etc. However, no e-waste activity is conducted in this community [47].

### Study participants

We recruited a total of two hundred and seven (207) participants, comprising one hundred and forty-two (142) informal e-waste workers and sixty-five [48] comparable subjects from Madina–Zongo into the study. At round I, we recruited one hundred and fifty-one (151) participants, comprising hundred (100) informal e-waste workers from Agbogbloshie and (fifty-one) 51 participants from Madina Zongo. Originally, recruitment was planned at wave I only, however, due to the high participant attrition of > 30% at wave II, new participants were enrolled (*n* = 42 and *n* = 14 for e-waste workers and the comparable population respectively), to replace those that were lost to follow-up. The study population comprised of male e-waste and a comparable population between the ages of 18 to 55 working at the Agbogbloshie e-waste site or resident at Madina Zongo. There are approximately 4500–6000 informal e-waste workers at the Agbogbloshie site who use rudimentary techniques and tools to recover valuable materials from waste electrical gadgets [49, 50]. A recent detailed study of their time-activity diaries revealed that e-waste workers work from 7:00 am to 6:00 pm at an average of 10 h per day, 6 to 7 days a week [41]. These workers are engaged in a myriad of job-specific tasks. The major job tasks include collecting and sorting e-waste.

### Data collection

We measured PM (PM_10_, and PM_2.5_) in the breathing zone of each participant using a real-time optical pump (Aerocet 831, Met One Instruments, Inc., Oregon, USA). We also performed 5-min resting electrocardiograms (ECG) for each participant according to a standardized protocol (Task Force of The European Society of Cardiology and The North American & Society of Pacing and Electrophysiology, 1996) to evaluate HRV. A questionnaire was also administered to elicit information on socio-demographic and other selected covariates. Three rounds (I, II, and III) of panel data collection were aligned to the prevailing weather patterns (dry, rainy, harmattan seasons) in the study areas. The comprehensive organizational procedures are documented elsewhere [41].

#### Interview with a structured questionnaire

At enrolment, data on sociodemographic and economic factors (age, sex, religion, ethnicity, education, occupation, socioeconomic and marital status), detailed current and previous job history and exposures; home exposures including indoor cooking and biomass fuel use, pre-existing medical conditions, and lifestyle habits such as cigarette smoking and alcohol use were collected using a comprehensive questionnaire. Data on cigarette smoking included age at smoking initiation, duration of smoking, sticks smoked per day, current or ex-smoker defined as smoking cessation greater than a month’s duration. Smokers were classified as current, ex-, or never–smokers. The questionnaire was administered by trained interviewers in a local dialect, Dagbani or Twi, or English according to the participant’s preference. Limited interview guides were administered in subsequent follow-up data collection that explored possible changes in home and work exposures, and other cardiovascular and respiratory conditions.

#### Personal particulate matter monitoring

We measured personal particulates (PM_10_, PM_10–2.5_, and PM_2.5_) in the breathing zone of each participant continuously per minute using a real time optical counter at a flow rate of 2.83 L/min. Personal level ambient temperature, relative humidity and barometric pressure were also measured every 1 min using logging instrumentation (UX100–003 data logger; Onset Corporation, Bourne, MA, USA). All the measuring instruments were carried in a customized backpack with inlets in the breathing zone of participants for 4 h of the working day, usually between 8 am and 2 pm. As a quality control measure, PM concentrations were considered invalid when TSP exceeded 2000 μg/m^3^ [38]. During round III however, the sampling duration was reduced to approximately 2-h due to the high levels of PM measured from PM laden (dust) harmattan winds.

#### Heart rate and heart rate variability measurement

Heart rate variability is the physiological phenomenon of the variation in the time interval between consecutive heartbeats in milliseconds. Another name used for HRV is the ‘R-R variability’, where R is the point corresponding to the peak of the QRS complex (Fig. S1) of the ECG wave. The term ‘NN’ is used in place of ‘RR’ to show the ‘normal’ to ‘normal’ beats on the ECG (Task Force of the European Society of Cardiology and the North American Society of Pacing and Electrophysiology 1996). The QRS complex is the combination of three of the graphical deflections seen on a typical ECG. For this study we measured resting ECG using a 12-channel digital ECG recorder (Sonoscape Medical Corp, Shenzen, China), a continuous medical test device that measures heart rate, rhythm, and other abnormalities that may affect normal heart function, according to standard procedure [51, 52]. HRV was measured with a variation in the RR intervals exhibited in a sequence of ECG sample using the short-term measurement for 5 min under standard conditions. After a 5–10 min rest, each participant’s ECG was recorded (sampling rate of 256 Hz per channel) for approximately 5 min with the subject lying supine and still. We used the best 5-consecutive-minute interval for the HRV calculations. The ECG digital recordings were processed, and heart rate and HRV indices were assessed using PC-based software, Kubios HRV premium software version 3.3 (Kubios HRV Software, Kuopio; Finland). Beats were automatically detected and assigned tentative annotations, which were then reviewed by an experienced technician to correct for any mislabeled beats or artifacts. HRV was assessed through time and frequency domain variables. Only normal-to-normal (NN) beat intervals were included in the analysis. We computed standard deviation of NN intervals (SDNN), the square root of the mean of the squared differences between adjacent NN intervals (RMSSD), high-frequency power (HF) (0.15–0.4 Hz), and low-frequency power (LF) (0.04–0.15 Hz). We also estimated the LF/HF ratio to show the relative balance between the sympathetic and the vagal nerve activity [14]. HRV was analyzed when RR intervals were ≥ 90% of all R-R intervals [53].

### Ethical clearance

Ethical clearance for the study was obtained from the ethical and protocol review committee of the College of Health Sciences (CHS-Et/M.4-P 3.9/2015–2016), University of Ghana and the University of Michigan’s Institutional Review Board (IRB). Recruited participants provided a written informed consent endorsed by signature or thumbprint. Permissions were also obtained from the e-waste association and community leaders of the study sites.

### Statistical procedures

Data were analyzed using the statistical package STATA version 15 (Statacorp LLC, College Station, TX, USA**)**. A descriptive analysis of the characteristics of the study participants was based on data obtained through the baseline questionnaire. Independent sample t-test was applied for the two-group comparisons of normally distributed data and the Mann Whitney U test to the two group comparisons of non-normally distributed data. We used repeated measure Analysis of variance (ANOVA) to compare the HRV, HR and blood pressure indices among the e-waste workers to the controls. We observed significant reductions in all the Log_10_ transformed HRV indices (i.e., SDNN, RMSSD, Mean NN, LF, HF and ratio of LH/HF) for e-waste workers compared to controls across all three sampling periods (rounds I-III). Measures of HRV indices were log_10_-transformed to improve normality and stabilize variances for suitability to analyze data by mixed linear regression [54]. Mixed models with a random participant effect and covariance structure were used to analyze the association between air pollutants (PM _10_, PM_10–2.5_ and PM_2.5_) and the ECG parameters considering the repeated measurements over time for each individual. We further included a linear term of each HRV measure in the model. The models were adjusted for age, BMI, job category, alcohol use, cigarette smoking, ambient temperature, barometric pressure, and relative humidity. Models were built for each ECG parameter separately. For effect modification, we considered body mass index (BMI > 25.0 kg/m^**2**^, smoking history (ex-smokers vs. never-smokers) and alcohol use. Effect estimates are presented as percent change from the mean (arithmetic mean for all other ECG-variables) together with 95%-confidence intervals based on an interquartile range increase in PM concentration. All the analyses were performed using STATA® version 15 software. *P* <  0.05 was considered statistically significant.

## Results

### Sociodemographic and health characteristics of study participant

As shown in Table 1, the participants were men with mean (SD) age of 27.87(8.14) years and ranged from 16 to 55 years. The comparable population was on average older (30.97 **±** 9.92 yrs. vs 26.45 **±** 6.74 years). More than half, 77(55%), of e-waste workers either had no formal or primary school education as compared to 46 (62%) of the comparable population with high school education. Mean age, daily income, and educational level were significantly lower among the e-waste workers than the comparable population. Average body mass index (BMI) was 23.19 ± 3.05 kg/m^2^ and 23.75 ± 3.44 kg/m^2^ for e-waste workers and comparable population respectively, with no significant differences in BMI between the e-waste workers and comparable group (*p* = 0.100). The mean ± SD systolic and diastolic blood pressure among the study participants was 124.34 ± 15.52 mmHg and 74.13 ± 10.42 mmHg respectively. When the blood pressure parameters were categorised based on the 2017 American Heart Association Hypertension guidelines, 94 (66.2%) e-waste workers and 35(54.7%) comparable group had normal blood pressure; whereas 14 (21.9%) e-waste workers and 18 (12.7%) individuals of the comparable group had elevated blood pressure. 11(17.2%) e-waste workers versus 17(12.0%) comparable group, and 4 (6.3%) e-waste workers versus 13(9.2%) comparable group (*p* = 0.198) were in the stages 1 and stage 2 hypertension respectively. The frequency of cigarette smoking among e-waste workers and comparable group was 40 (25%) and 6 (10%) respectively (*p* <  0.001). Prevalence of pre-existing cardiovascular conditions including hypertension 3(4.0%) among e-waste workers compared to 8 (19.1%) among the non-e-waste workers (*p* ≤ 0.001). Prevalence of diabetes mellitus among the e-waste and comparable population were 1(1.40%) versus 4 (9.50%); (*p* = 0.005) respectively. None of the e-waste workers reported a previous history of dyslipidaemia compared to 5 (11.90%) among the comparable population (*p* < 0.001). There were also observable differences in the personal ambient barometric pressure between e-waste workers and comparable group [i.e., 750.89 ± 33.6 mmHg for e-waste workers versus 956.97 ± 105.09 mmHg for comparable group; (*p* = 0.014). Similar observations were made for ambient temperature [i.e., 37 ± 2.21 °C for e-waste workers versus 35.00 ± 3.49 °C for non-e-waste workers (*p* < 0.001). However, relative humidity levels between the e-waste workers and comparable group were not significantly different [i.e., 56.13 ± 5.51% for e-waste workers versus 60.91 ± 9.55% among comparable population (*p* = 0.229).

**Table 1:** Socio-demographic and health Characteristics of study participants

**Variable**	**Total (*N*** **= 207)**	**E-waste Workers** **(*N*** **= 142)**	**Comparable group (*N*** **= 65)**	***P*-value**
**Age in years: mean ± SD**	27.87 **±** 8.14	26.45 **±** 6.74	30.97 **±** 9.92	**< 0.001**
**Education** (%)				**< 0.001**
None	45(22.96)	39(27.86)	6(10.71)	
Primary	42(21.43)	38(27.14))	4(7.14)	
Middle/JHS	61(31.12)	44(31.43)	17(30.36)	
Secondary/SHS+	48(24.49)	19(12.86)	29(31.29)	
**Marital Status**				**0.05**
*Single*	74(49.0)	44(45.4)	30(58.8)	
*Married*	74(49.0)	53(54.6)	21(41.2)	
**Daily Income** (%)*				**< 0.001**
≤ GHS 20	36(18.00)	26(18.44)	10(16.95)	
GHS 20–40	59(29.5)	46(32.62)	13(22.03)	
GHS 41–60	57(28.5)	38(26.95)	19(32.2)	
GHS 61+	48(24.0)	31(20.91)	16(3.28)	
**Indoor cooking** (%)	45(22.73)	22(15.94)	23(38.33)	**< 0.001**
**Lifestyle habits** (%)				
**Alcohol use** (%)				0.181
Current	24(11.94)	19(13.48)	5(8.33)	
Previous	7(3.48)	3(2.13)	4(6.67)	
Never	170(84.58)	119(84.40)	51(85.00)	
**Cigarette smoking** (%)				**0.001**
Current	50(25.13)	27(41)	6(10)	
Never	149(74.87)	96.2(111)	54(90)	
**4-h personal air weather variables**				
Relative humidity (%)	57.59 ± 7.32	56.13 ± 5.51	60.91 ± 9.55	0.229
Barometric pressure (mmHg)	7410.79 ± 63.40	7512.89 ± 33.6	7170 ± 105.09	**0.037**
Ambient temperature (°C)	36.49 ± 2.84	37.15 ± 2.21	35.00 ± 3.49	**< 0.001**
**Self-reported medical History** (%)				
Pulmonary Tuberculosis	2(1)	2(1.42)	0(0)	0.86
Bronchial Asthma	7(3.48)	5(3.55)	2(3.33)	**0.010**
Hypertension				**0.001**
Yes	11(9.3)	15.2(12)	8(19.1)	
No	90(76.3)	84.6(66)	33(78.6)	
Diabetes Mellitus				**0.005**
Yes	5(4.3)	2.6(2)	4(9.5)	
No	91(78.5)	97.4(75)	36(85.7)	
Dyslipidaemia				**0.000**
Yes	5(4.3)	2.6(2)	5(11.9)	
No	89(76.1)	94.9(74)	35(83.3)	
Don’t know	23(19.7)	2.6(2)	2(4.8)	
**Medications** (%)				
Anti-hypertensive				0.730
Yes	7(18.4)	5(20)	2(15.4)	
No	31(81.6)	20(80)	11(84.6)	
Anti-diabetes				0.540
Yes	2(5.7)	1(4.2)	1(9.1)	
No	33(94.3)	23(95.8)	10(90.9)	
Statins				
Yes	2(6.1)	2(8.7)	0(0)	1.000
No	31(93.9)	21(91.3)	10(100)	
	**Total**	**E-waste (*n*** **= 100)**	**Comparable group (***n* = **42)**	***P*-value**
**BMI (kg/m**^**2**^**): mean ± SD**	23.37 ± 3.19	23.19 ± 3.05	23.75 ± 3.44	0.100
**BMI category (n, %)**				
**Normal - BM1 18-25 kg/m**^**2**^**, (n, %)**	161(77.8)	117(83.39)	44 (67.69)	0.404
**Abnormal - BMI > 25 kg/m**^**2**^**(n, %)**	46 (22.22)	25(17.61)	25 (32.31)	0.113
**Blood pressure (mmHg): mean ± SD**				
Systolic blood pressure	124.34 ± 15.52	122.31 ± 12.62	128.84 ± 17.58	**0.010**
Diastolic blood pressure	74.13 ± 10.42	73.15 ± 9.31	75.98 ± 12.55	0.110
**High Blood pressure category (n, %)**				
Normal	129(62.6)	94(66.2)	35(54.7)	0.198
Elevated	32(15.5)	14(21.9)	18(12.7)	
Stage 1	28(13.6)	11(17.2)	17(12)	
Stage 2	17(8.3)	4(6.3)	13(9.2)	
**Resting pulse rate (bpm):** mean ± SD	74.1 ± 12.1	74.2 ± 12.6	74.1 ± 13.6	0.960
**Resting pulse rate category (n, %)**				
< 85 bpm (Normal)	157(75.85)	107(75.35)	50(76.92)	0.806
≥ 85 bpm (Abnormal)	50(24.15)	35(24.65)	15(23.08)	
**Blood oxygen saturation (%)**: mean ± SD	96.56 ± 3.23	96.34 ± 3.64	97.06 ± 1.94	**0.019**
**Blood oxygen saturation category (n, %)**				
≥ 95% (Normal)	94.7 (195)	93.7 (133)	96.9 (62)	0.343
< 95% (Abnormal)	11(5.3)	9(6.3)	2(3.1)	
**Heart rate variability (Mean ± SD)**				
Log LF	10.4 ± 1.73	11.1 ± 0.74	10.0 ± 2.01	**≤0.001**
Log HF	9.8 ± 2.03	9.2 ± 2.20	10.8 ± 0.92	**≤0.001**
LH/ HF ratio	2.4 ± 2.23	2.8 ± 2.32	1.8 ± 1.40	**0.004**
Log SDNN	5.43 ± 0.61	5.2 ± 0.71	5.8 ± 3.02	**≤0.001**
Log RMSSD	5.21 ± 0.81	4.9 ± 0.81	5.7 ± 0.30	**≤0.001**
MeanNN	5.82 ± 1.91	5.9 ± 0.33	6.4 ± 0.21	**≤0.001**

Student t-test and Pearson Chi-square comparing the means and proportions between e-waste workers and a comparable population from Madina Zongo

Abbreviations: SpO_2 –_ blood oxygen saturation. BMI- body mass index (kg/m^2^). PM, relative humidity, barometric pressure, and ambient temperature were sampled for 4- h of the workday. Bold *p*-values represent significant values (*p* ≤ 0.05). Variations in health outcome characteristics among e-waste and comparable population. Hypertension classification is based on 2017 AHA guidelines. *Stage 1: 130–139/80-89 mmHg; Stage 2: ≥140/≥90 mmHg*

Abbreviations: SpO_2 **–**_ blood oxygen saturation. BMI- body mass index (kg/m^2^) Mean NN- mean normal to normal beats; SDNN- standard deviation of normal-to-normal; RMSSD; LF - low frequency; HF – high frequency; LF/HF ratio- the ratio of high frequency and low frequency. Bold *p*-values represent significant values (*p* ≤ 0.05)

*1US dollar (USD) is approximately 5.83 Ghanaian Cedi

### Variations in particulate matter concentrations in personal air of study participants

The levels of PM_2.5_, PM_10–2.5_ and PM_10_ at each sampling period is shown in [Table S1]. The levels of PM_2.5_, PM_10–2.5_ and PM_10_ across the study period increased at each round with the highest concentrations recorded during the third sampling round (from Jan to March 2018), corresponding to the harmattan season (a period when dry and dust laden winds blows along the northwest coast of Africa from the Sahara Desert). The median/IQR concentrations of PM (2.5, 10–2.5 and 10 μm) among e-waste workers and comparable population (Table S1) over study time were consistently higher than the WHO air quality standards of 25 μg/m^3^ and 50 μg/m^3^ per 24-h mean. Personal PM_2.5_ concentrations among e-waste workers for rounds I, II and III were 69.86 ± 36.33 μg/m^3^, 61.18 ± 37.92 μg/m^3^ and 70.69 ± 48.25 μg/m^3^; that of the comparable group were 34.88 ± 14.72 μg/m^3^, 34.13 ± 7.22 μg/m^3^ and 50.21 ± 169.64 μg/m^3^ respectively. There were significant differences in concentrations of PM_2.5_ by rounds between the two groups; (I) *p* < 0.001, (II) *p* < 0.001 and (III) *p* = 0.950 respectively. Although the levels of PM_2.5_ was high at both study sites, twice as high concentrations of PM_2.5_ were recorded in the breathing zone of e-waste workers, relative to the comparison group. Similar trends were observed for the PM_10–2.5_ fraction across the study period I to III [i.e. 94.26 ± 87.34 μg/m^3^ versus 68.23 ± 63.31 μg/m^3^, *p* = 0.009]; [48.88 ± 84.68 μg/m^3^ versus 34.74 ± 55.41 μg/m^3^, *p* = 0.009] and [54.30 ± 126.38 μg/m^3^ versus 31.59 ± 383.81 μg/m^3^, *p* = 0.960] respectively. Also, higher levels of PM_10_ were consistently observed among e-waste workers than comparable group [(214.43 ± 154.46 μg/m^3^ vs. 118.12 ± 79.99 μg/m^3^, *p* ≤ 0.001), (173.49 ± 96.12 μg/m^3^ vs. 99.35 ± 107.1 μg/m^3^; *p* ≤ 0.001), and 181.64 ± 104.46 μg/m^3^ vs. 395.57 ± 412 μg/m^3^, *p* = 0.998)]. Description of personal levels of PM among study participants is already published [40].

### Variations in heart rate variability indices, and resting heart rate among study participants

Comparison of HRV indices among study participants is presented in [Table 2]. These values are the means (SD) of the test results for each individual, averaged over the round of the study period. Mean values are presented showing the differences between the informal e-waste workers and the comparable population during rounds I and II, there was significant evidence of reduction in log_10_ SDNN among e-waste workers compared to the comparable group [(5.23 ± 0.68 vs 4.88 ± 0.08), *p* = ≤ 0.001, and (5.28 ± 0.67 vs. 5.60 ± 0.55), *p* = ≤ 0.001]. However, during round III, the control population showed a lower mean log_10_ SDNN compared to the e-waste workers (5.02 ± 0.82 vs 5.34 ± 0.67), *p* = 0.030. The results of our study also showed changes in HRV indices at different seasons/weather conditions. Relative to the dry season, HF decreased in the rainy season whilst LF/HF increased in the harmattan among both e-waste and the comparison group. Among the e-waste workers RMSSD decreased whilst SDNN increased in both the rainy and harmattan season. The reverse was observed for the comparison group. Resting HR between e-waste workers and comparable group at round I was similar (73.91 ± 10.16 vs.74.09 ± 13.63), *p* = 0.960 and III (73.91 ± 10.16 vs 74.00 ± 12.67), *p* = 0.960, but controls had lower mean HR during round II (70.16 ± 10.64 vs. 69.52 ± 10.54), *p* = 0.050.

**Table 2:** Variation in Heart rate variability among e-waste and non-e-waste worker

Variable	E-waste –workers (***n*** = 142)	Comparable group (***n*** = 65)	***p***-value
**5-min resting Heart Rate Variability, Mean (SD)**			
**RI (Dry season)**			
Mean NN	5.97 ± 0.31	6.35 ± 0.21	**≤ 0.001**
Log SDNN	4.23 ± 0.68	5.88 ± 0.08	**≤ 0.001**
Log RMSSD	4.88 ± 0.84	5.72 ± 0.30	**≤ 0.001**
Log LF	9.98 ± 1.98	11.12 ± 0.68	**≤ 0.001**
Log HF	9.25 ± 2.25	10.77 ± 0.91	**≤ 0.001**
LF/HF ratio	2.75 ± 2.33	1.84 ± 1.66	**0.004**
**R II (Rainy season)**			
Mean NN	6.01 ± 0.29	6.24 ± 0.30	**≤ 0.001**
Log SDNN	5.28 ± 0.67	5.60 ± 0.55	**≤ 0.001**
Log RMSSD	4.85 ± 0.93	5.48 ± 0.74	**≤ 0.001**
Log LF	10.00 ± 2.07	10.71 ± 1.53	**0.030**
Log HF	9.19 ± 2.49	10.28 ± 1.83	**0.010**
LF/HF ratio	3.50 ± 5.12	1.85 ± 1.31	**0.010**
**R III (Harmattan season)**			
Mean NN	5.89 ± 0.26	5.91 ± 0.84	**0.841**
Log SDNN (ms)	5.02 ± 0.82	5.34 ± 0.67	**0.030**
Log RMSSD (ms)	4.55 ± 0.94	5.05 ± 0.83	**0.010**
Log LF (ms^2^)	9.36 ± 2.38	10.22 ± 1.71	**0.030**
Log HF (m/s)	8.45 ± 2.57	9.57 ± 2.06	**0.010**
LF/HF ratio	3.60 ± 2.93	2.46 ± 1.81	**0.010**
**Heart rate (beats/min), Mean (SD)**			
Round I	73.91 ± 10.16	74.09 ± 13.63	0.960
Round II	70.16 ± 10.64	69.52 ± 10.54	**0.050**
Round III	73.91 ± 10.16	74.00 ± 12.67	0.960

Sampling rounds were aligned to annual seasonal changes in study area, i.e. R I, II and III were aligned to the dry, rainy and harmattan respectively

Abbreviations: *R* round, *Mean NN* mean normal to normal beats, *SDNN* standard deviation of normal-to-normal, RMSSD; *LF* low frequency, *HF* high frequency, *LF/HF ratio* ratio of high frequency and low frequency

Bold *p*-values are significant at *p* ≤ 0.05

### Association between particulate matter exposure and heart rate variability indices

Following the conventional procedures in the HRV protocols, all HRV indices were log transformed. The primary theory in this study was that increasing concentrations of PM_2.5_, PM_10–2.5_ and PM_10_ levels in the breathing zone of e-waste workers is associated with decrease in HRV indices. The crude and adjusted estimates of PM exposure on HRV is presented in [Table 3]. The effect estimates for the association between PM_2.5_, PM_10–2.5_ and PM_10_ and the HRV indices (i.e. SDNN, RMSSD, LF, HF and HF/LF ratio) were mixed. The mixed effect model, accounting for time lags, showed minimal percent changes in HRV indices, even after adjusting for covariates and this varied according to different PM sizes. A negative association existed between PM2.5 and Mean NN for both crude and adjusted model and this association was statistically significantly. Adjusted model showed RMSSD and LF were negatively associated with PM 2.5, 10–2.5 and 10 but were not statistically significant. Results from the adjusted models showed that SDNN (the standard deviation of the normal-to-normal) was negatively associated with PM. A 10 μg/m^3^ per interquartile increase PM_10–2.5_ and PM_10_, decreased SDNN by 11% (95% CI: − 0.002- 0.000); *p* = 0.187 and 34% (95% CI: − 0.002-0.001); *p* = 0.035 respectively. However, PM_2.5_ increased SDNN by 34% (95% CI: − 1.32-0.64); *p* = 0.493 and the association was statistically insignificant. SDNN represents an estimation of the total HRV over a specified time (in milliseconds).

**Table 3:** Association between particulate matter exposure and heart rate variability indices

Variable	Crude		Adjusted	
Heart rate variability	β[95% CI]	***P-***value	β [95% CI]	***P-***value
**MeanNN**				
PM_2.5_	−0.54(− 0.99, − 0.097)	**0.017**	− 0.76(− 1.48, − 0.04)	**0.039**
PM_10–2.5_	0.01(− 0.12,0.12)	0.989	0.14(− 0.08,0.25)	0.218
PM_10_	0.05(− 0.09,0.19)	0.448	0.05(− 0.14,0.25)	0.591
**SDNN**				
PM_2.5_	− 0.35(− 0.71,0.01)	0.059	0.34(− 1.32,0.64)	0.493
PM_10–2.5_	0.63(0.11,1.13)	**0.017**	−0.11(− 1.73,0.95)	0.846
PM_10_	−0.52-0.95, − 0.93)	**0.017**	− 0.34(− 1.32,0.64)	**0.035**
**RMSSD**				
PM_2.5_	0. 49(− 0.86,0.33)	**≤0.001**	−0.27(−1.34,0.79)	0.620
PM_10–2.5_	0.93(− 0.41,1.46)	**≤0.001**	− 0.01(−1.26,1.28)	0.275
PM_10_	−0.77(− 0.86, − 1.24)	**0.009**	− 0.57(− 1.56,0.46)	0.255
**LF**				
PM_2.5_	−0.002(− 0.009,0.004)	0.507	− 0.007(− 0.004,0.023)	0.154
PM_10–2.5_	0.003(− 0.006,0.012)	0.552	− 0.006(− 0.101,0.002)	0.479
PM_10_	0.002(− 0.006,0.012)	0.533	− 0.004(− 0.021,0.010)	0.213
**HF**				
PM_2.5_	0.002(−0.005,0.010)	0.521	0.010(−0.005,0.025)	0.199
PM_10–2.5_	0.001(−0.002,0.002)	0.903	−0.004(− 0.009,0.001)	0.107
PM_10_	−0.001(− 0.004,0.001)	0.310	− 0.005(− 0.012,0.001)	0.104
**LF/HF**				
PM_2.5_	0.008(−0.002, 0.019)	0.117	0.020(−0.256,0.301	0.867
PM_10–2.5_	0.013(0.001, −0.245)	**0.035**	0.004(−0.402–0.032)	**0.030**
PM_10_	0.015(− 0.029–0.011)	**0.035**	0.003(−0.273–0.034)	0.836
**HR (beats/min)**				
PM_2.5_	0.014(−0.023, 0.051)	0.462	0.066(−0.015,0.147)	0.110
PM_10–2.5_	0.001(−0.007,0.009)	0.862	−0.001(− 0.027,0.025)	0.932
PM_10_	0.002(−0.013,0.009)	0.742	−0.010 (− 0.040,0.020)	0.522

Controlled for age, BMI (overweight), job category, smoking status, alcohol use, study location, and weather variables (ambient temperature and relative humidity)

Bolden *p*-value is significant at *p* ≤ 0.05

Also, a 10 μg/m^3^ increase in PM_2.5_, PM_10–2.5_ and PM_10_ decreased RMSSD by 27% [(− 1.34–0.79); *p* = 0.620, 11% (− 1.73, 0.95); *p* = 0.846 and 0.57% (− 1.56–0.46); *p* = 0.2551] respectively after adjusting for selected covariates. However, the associations were not significant. RMSSD estimates short-term (high frequency) components of HRV which reflects the relative influence of the parasympathetic nervous system. In the adjusted models, a 10 μg/m^3^ increase in PM_2.5_, PM_10–2.5_ and PM_10_ range, decreased Mean NN by 76% (95% CI: − 1.48, − 0.04); *p* = 0.039. The association was statistically significant. However, PM_10–2.5_ and PM_10_ increased Mean NN by 14% (95% CI: − 0.08-0.25); *p* = 0.218 and 5.0% (95% CI: − 0.09- 0.19); *p* = 0.591 respectively.

After adjusting the model for selected covariates, PM_2.5_, PM_10–2.5_ and PM_10_ decreased LF by 0.7% (95% CI:--0.004–0.023); *p* = 0.154, 0.6% (− 0.101–0.002); *p* = 0.479 and 0.4% (95% CI: − 0.021-0.010), *p* = 0.213 respectively. In the adjusted model, PM_2.5_, increased HF by 1.0% (95% CI:--0.005–0.025); *p* = 0.199), whilst both PM_10–2.5_ and PM_10_ decreased HF by 0.4% (95% CI -0.009-0.001); *p* = 0.107) and 0.5% (95% CI:-0.012–0.001); *p* = 0.104). For every 10 μg/m^3^ per interquartile increase in PM_2.5_, the LF/HF ratio increased by 6.6% (95% CI:-0.015–0.147); *p* = 0.110. PM_10–2.5_ and PM_10_, however decreased LF/HF ratio by 0.1 (− 0.027–0.025); *p* = 0.932 and 1.0% (95% CI:-0.040–0.020); *p* = 0.522.

### Effects of selected covariates on the association of PM exposure on HRV indices

The associated effects of PM exposure and selected covariates is presented in [Table S2]. E-waste Job tasks were negatively associated with all HRV indices. A 10 μg/m^3^ increase in PM decreased RMSSD by 98% (95% CI: − 1.62, − 0.33), 64% (95% CI: − 1.28, − 0.01), 62% (95% CI: − 0.94,-0.29) and 59% (95% CI: − 0.96, 0.21) among e-waste collectors, sorters, dismantlers and burners respectively and these associations were highly significant (*p* ≤ 0.001; *p* ≤ 0.001; *p* = 0.05; p ≤ 0.001). SDNN were equally reduced by 37% (95% CI: − 0.82, 0.08) among collectors, 34% (95% CI: − 0.61, − 0.08) among burners, 29% (95% CI: (− 0.53, 0.07) among dismantlers, and 29% (95% CI: (− 0.73, 0.16) among sorters. LF was also reduced across all job categories except among sorters, where we observed an increase of 26% (95% CI: − 1.33, 1.85); *p* = 0.75. Among burners, collectors and dismantlers, LF reduced by 114% (95% CI: − 2.08, − 0.19), *p* = 0.02; 94% (95% CI: − 1.74, − 0.13), *p* = 0.02 and 63% (95% CI: − 2.24, 0.98), *p* = 0.04 respectively. HF values were reduced across all e-waste categories. HF decreased among burners by 126% (95% CI: − 2.35, − 0.16), *p* = 0.03; 99% (95% CI: − 1.92, − 0.06), *p* = 0.04] among dismantlers, 15% (95% CI: − 1.99, 1.69), *p* = 0.88 among sorters and 163% (95% CI: − 3.49, 0.23), *p* = 0.09 among sorters. The relationship of HRV indices with age was variable. Generally increased age is associated with a marginal increased in RMSSD [(95% CI: − 0.01, 0.02); *p* = 0.58] and SDNN [(95% CI: − 0.01, 0.01); *p* = 0.97], by 1.0% and decreased LF [(95% CI: − 0.04, 0.04); *p* = 0.97] and HF [95% CI: − 0.05, 0.05); *p* = 0.96] by 1.0%. Overweight (BMI > 25 kg/m^2^) was associated with decreases in RMSSD, SDNN, LF and LF [i.e. 16% (95% CI: − 0.47, 0.16), *p* = 0.33; 11% (95% CI: − 0.33, 0.12), *p* = 0.34; 64% (95% CI: − 1.43, 0.15),*p* = 0.11; 27% (95% CI: − 1.18, 0.65), *p* = 0.57) respectively]. The associations were insignificant. Indoor cooking was also associated with negative but insignificant associations with HRV. Indoor cooking reduced RMSSD by 1% (95% CI: − 0.32, 0.31); *p* = 0.97, SDNN by 4% (95% CI: − 0.26, 0.18): *p* = 0.72, LF by 13% (95% CI: − 0.91, 0.66); *p* = 0.75 and HF by 18% (95% CI: − 0.72, 1.09); *p* = 0.69).

## Discussion

Occupational exposure to PM has been associated with adverse cardiovascular health outcomes. It is established that both acute and chronic exposures to PM are related to CVDs [43]. We conducted a longitudinal cohort study that assessed the association of personal ambient PM_2.5_, PM_10–2.5_, and PM_10_ on the cardiac autonomic function of healthy informal e-waste workers. We observed consistent reductions in HRV, and HR across the sampling periods due to the direct exposure to PM (2.5, 10–2.5 and 10 μm) among the informal e-waste workers. We observed a consistently decreased HRV indices (when values were log_10_ transformed over time and frequency) among informal e-waste workers across the three sampling periods compared to the control group. In evaluating the percentage change of PM (2.5, 10–2.5 and 10 μm) exposure on HRV over time, we observed a non- significant decrease in HRV among e-waste workers of different job task after adjusting the models for covariates such as age, sampling location, sampling round, personal ambient temperature, barometric pressure and relative humidity. *Other physical and chemical work-related factors such as noise, chemicals (*e.g. *carbon disulfide, lead, manganese and organic solvents, such as n-hexane, xylene, and toluene) and radiation, present in the e-waste recycling environment may also adversely affect the cardiovascular and autonomic nervous systems, thereby resulting in fluctuations in heart rate and HRV* (Aarbaoui & Chaix, 2019; Health Protection Agency, 2012; Shokr, 2015; Sim et al., 2015; TOGO & Takahashi, 2009)*). The possibility of these occupational factors interacting or confounding the effect of PM on HRV exist* (Huang et al., 2013)*. However, these factors were not measured in this study and their effect on PM and HRV was not explored. Future studies will focus on addressing that.*

The influence of the cardiac autonomic nervous system in balancing the cardiovascular system during rest and work can be assessed with HRV and, in occupational health, this is commonly describe using the frequency domain and time domain HRV parameters (e.g. RMSSD, HR Mean NN, LF, HF, SDNN) [55]. RMSSD reflects the beat-to-beat variance in HR and is the primary time-domain measure used to estimate the vagal nerve -mediated changes reflected in HR. Mean NN measures the variations in R-R intervals over time and the response of the CVS to diverse environmental stimuli and workloads; an abnormal beat reflects cardiac dysfunction. The LF band (0.04–0.15 Hz) is typically recorded over a minimum of 2 min period and reflects the respiratory-related efferent vagal - mediated influences. HF is a frequency-domain HRV statistic that summarizes the power spectral density of the heartbeat from 0.15 to 0.4 hertz and is an estimator of parasympathetic autonomic control. A low LF/HF ratio indicates high parasympathetic dominance; in contrast, a high LF/HF ratio reflects sympathetic dominance, which occurs in fight-or-flight situations. The SDNN, which shows the10-second as well as the fast fluctuations in heart rate, reflects the sympathetic and parasympathetic involvement.

The reduction in HRV indices among participants may be as a result of external factors (such as climate conditions, job related conditions, exposure harmful substances), lifestyle habits, diseases and physiological factors as shown in another study [56]. The variation in PM sizes, possibly from different sources, and season may be considered as factors contributing to the changes, although small, observed in HRV indices when association between HRV indices and lag PM and seasons were evaluated. The study evaluated the effect of some of these factors (identified as covariates) on HRV and results showed a reduction in HRV. The consistent reduction in HRV parameters among e-waste workers at each sampling period demonstrates that they have less favorable health and an increased risk of arrhythmias [57] than comparable group and this may be linked to their job task and exposure to PM generated as shown by covariate analysis. The high reduction of RMSSD among e-waste collectors may partly be explained by the potential exposure to multiple pollutants. Collectors typically trek around town, scavenging for scrubs and wasted EEEs. Concurrent exposure to vehicular exhaust particles, smoke from biomass, entrained dusts and other sources of PM be accountable for the observed results.

Studies reporting PM-associated reductions in HRV have been conducted among elderly populations and patients with primarily lung diseases such as COPD [58] and asthmatics [59, 60]. PM-associated reductions in HRV effects have been associated with all PM fractions, although some inconsistencies between the studies do exist. Albeit this, some inconsistencies have been observed in studies among both young and healthy subjects in terms of the direction of the HRV changes [1, 61, 62].

Previous occupational studies have shown a negative association between increased PM_2.5_ exposure and variations in the autonomic function as indicated by changes in HRV (measured in time and frequency domains). In most of the studies, measures of HRV were reduced, suggesting an overall trend toward withdrawal of parasympathetic tone. For example, Wilson and colleagues [30], examined the associated effects of PM_2.5_ exposure on HRV indices among restaurant and bar workers (*n* = 14). They found that for every 10 μg/m^3^ increase in PM_2.5_ decreased SDNN by 2.66% and RMSSD by 3.79%. In our study recorded similar findings except that the SDNN and RMSSD percentage decreases were greater, thus, 10 μg/m^3^ per interquartile increase in PM_2.5_ increased SDNN decreased by 35% and RMSSD by 27.0%. Also, they observed an increase in post shift HR to 108 beats/min. From baseline. In a cross sectional study among vehicle maintenance workers (*n* = 5) who were exposed to PM_2.5,_ Eninger & Rosenthal [63] reported decreases in SDNN, TP, RMSSD and HF with increasing PM_2.5,_ but the associations were statistically insignificant except for total spectral power (*p* < 0.05). We observe marginal decreases in HF of 1.0% (*p* = 0.199). In another panel study conducted among boiler construction workers (*n* = 36) exposed to PM_2.5_ in welding fumes in the United States of America high concentrations of metal component of PM_2.5_ in welding fumes was associated with reduced night time RMSSD [19].

In a longitudinal cohort study among boiler construction workers (*n* = 39) who were exposed to high PM_2.5_ concentrations (mean 697 μg/m^3^), it was found that 1 mg/m^3^ increase in PM_2.5_ was associated with 2.26% decrease in 5-min SDNN and 1.02% increase in HR [27], and correlation between PM_2.5_ and SDNN was statistically significant. Their observation may be attributed to the high average PM exposures, which were over 10 times greater than the levels observed in this study. Riediker [61] also demonstrated decreases in HR among highway patrol troopers exposed to PM in their cars, while subjects exposed aboard a diesel bus also experienced increased HR. [1] Some epidemiological studies have reported immediate cardiovascular system responses, within hours of exposure to PM [12], but more often same day delayed responses were detected [22, 48, 64, 65], suggesting that PM - induced effects on autonomic function seem to accumulate in association with prolonged elevated air pollution.

Because of the small sample size in this study, and the relatively low level of exposure, no definitive conclusion could be drawn about the relationship of occupational exposure to PM_2.5_ and HRV in normal subjects. In addition, this study, as in many of the previous studies, current study did not further investigate the chemical composition of PM_2.5_. Particulate matter composition differs by source and some are more toxicologically active than others. Additionally, aerosol composition likely varies between work, transit, ambient, and home environments. Initial research has suggested an association of HRV with the metallic component of PM_2.5_ in the workplace [27, 29]. In comparing occupational studies of PM_2.5_ and HRV in worker populations with those done in the general population, these differences, as well as the differences in the age distribution and other characteristics of the respective populations, must be considered. Further studies of the effects of workplace PM_2.5_ on HRV should examine the role of exposure level and exposure composition in larger and more varied worker populations.

## Conclusion

Overall, our study is consistent with several epidemiological studies which support the hypothesis that ambient concentrations of PM represent an environmental stressor that could alters autonomic balance, and subsequent increased risk for cardiovascular diseases including ischemia, arrhythmias, or myocardial infarctions. Specifically, our study showed that personal ambient particulate matter exposure is significantly associated with HRV. This suggests that exposure to ambient PM could have adverse health effect on cardiovascular function.

### Strength and limitations of study

As far as we know, this is the first longitudinal study examining the effects of e-waste recycling associated PM in the breathing zone of workers on workers’ HRV in occupational environment. Second, we measured real-time personal air samples for the concentration of PM in the breathing zone of the e-waste workers; which gives a more accurate measurement. This method is not used routinely due to the fact that personal monitoring pumps could become a hindrance to the subjects’ work tasks. We overcome this challenge with the use of light weight optical particle counter pumps assembled in customized backpacks, with inlets in the breathing zone, which could be worn comfortably by participants.

Performing real time ECG in an informal work environment is difficult due to noise, and inappropriate environmental constraints. Despite several logistics and space limitation, a temporary onsite clinic was erected to create the actively performed real time ECG to assess cardiac function among e-waste workers.

Our study also had some limitations. Ambient PM was the only contaminant sampled in this study. Other contaminants present in the air that may have an effect on HRV such as carbon monoxide, nitrous oxide and ozone were not measured. Basic health questions were asked of each subject participating in the study; however, none of the subjects underwent a baseline physical examination. Therefore, we are not able to rule out undiagnosed medical conditions, such as heart disease, which may have affected the results. Previous studies have found beta-blockers increase HRV in patients following heart attacks [66, 67]. Since most of our participants were young healthy adults, data was not collected on beta blocker drug use. Future studies should examine this possibility and carefully consider the use of beta-blocker medication as a potential confounder in assessing the effect of particulates exposures on HRV among elderly hypertensive e-waste workers.

Finally, noise is known to be negatively associated with HRV [68]. Background noise levels were not measured; however, the temporary onsite clinic environment was conducive for reducing interferences due to background noise level.

## Supplementary Information

**Additional file 1: Fig. S1.** A typical ECG tracing showing the QRS complex. **Table S1.** Variation in particulate matter concentrations between informal e-waste recyclers and the comparison population at Agbogbloshie, Accra- Ghana. **Table S2.** Associations of selected covariates on the association of PM exposure on HRV indices.

## Data Availability

The datasets used and/or analysed during the current study are not publicly available due to privacy issues but are obtainable from the corresponding author on reasonable request.

## Abbreviations

HRV: Heart rate variability
HF: High frequency power
LF: Low frequency power
LF/HF: Ratio of low frequency to high frequency power
RMSSD: Root-mean square of successive differences
SDNN: Standard deviation of normal RR intervals
BP: Blood pressure
SBP: Systolic blood pressure
DBP: Diastolic blood pressure
