# Supplementary material for: Personal exposure to particulate matter and heart rate variability among informal electronic waste workers at Agbogbloshie: a longitudinal study
Source: BMC Public Health. 2021 Nov 25;21:2161. doi: 10.1186/s12889-021-12241-2 (PMC8613947; doi:10.1186/s12889-021-12241-2)
Supplement: Supplementary file 1 — Additional file 1: Fig. S1. A typical ECG tracing showing the QRS complex. Table S1. Variation in particulate matter concentrations between informal e-waste recyclers and the comparison population at Agbogbloshie, Accra- Ghana. Table S2. Associations of selected covariates on the association of PM exposure on HRV indices. [file 12889_2021_12241_MOESM1_ESM.docx]

**Supplementary tables**

**Personal exposure to particulate matter and heart rate variability among informal electronic waste workers at Agbogbloshie: a longitudinal study.**

*Afua A. Amoabeng Nti^1^, Thomas G. Robins^3^, John Arko-Mensah^1^, Duah Dwomoh^2^, Lawrencia Kwarteng^1^, Sylvia Takyi^1^, Augustine Acquah^1^, Niladri Basu^4^, Stuart Batterman^3^, Julius N. Fobil^1^

1. Department of Biological, Environmental & Occupational Health Sciences, School of Public Health, University of Ghana; P.O. Box LG13, Accra, Ghana.
2. Department of Biostatistics, School of Public Health, University of Ghana; P.O. Box LG13, Accra, Ghana.
3. Department of Environmental Health Sciences, University of Michigan, 1415 Washington Heights, Ann Arbor, MI 48109, USA.
4. Faculty of Agricultural and Environmental Sciences, McGill University, Montréal, QC H9X 3V9, Canada.

**Fig. S1: A typical ECG tracing showing the QRS complex**


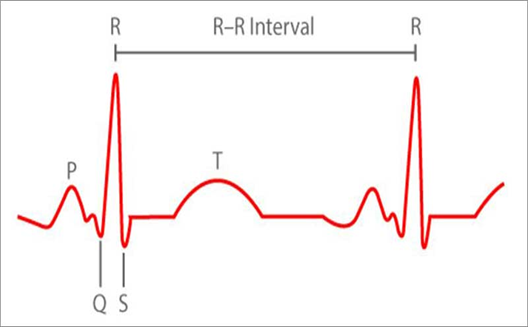


As shown in Fig. 1, QRS complex is the combination of three of the graphical deflections seen on a typical ECG. The QRS complex corresponds to the depolarization of the right and left ventricles of the human heart and contraction of the large ventricular muscles. The instantaneous heart rate (R-R interval) can be calculated from the time between any two QRS complexes. The P wave indicates atrial depolarization. It occurs when the sinus node creates an action potential that depolarizes the atria. The T wave occurs after the QRS complex and is a result of ventricular repolarization

Fig. 1: A typical ECG tracings showing the QRS complex (Wikimedia.org).

**Table S1: Variation in particulate matter concentrations between informal e-waste recyclers and the comparison population at Agbogbloshie, Accra- Ghana.**

| **Variable** | **Total (N=207)** | **E-waste Workers**  **(n=142)** | **Comparable population**  **(n=65)** | ***P*-value** |
| --- | --- | --- | --- | --- |
| **4- hour Personal PM concentration (µg/m^3^)** |  |  |  |  |
| **PM_2.5_ (fine)** |  |  |  |  |
| Round I | 59.06±43.89 | 69.86±36.33 | 34.88±14.72 | **<0.001** |
| Round II | 54.30±37.47 | 61.18±37.92 | 34.13±7.22 | **<0.001** |
| Round III | 69.67±61.23 | 70.69±48.11 | 50.21±169.64 | 0.950 |
| **PM_10-2.5_ (coarse)** |  |  |  |  |
| Round I | 87.48±75.86 | 94.26±87.34 | 68.23±63.31 | **0.009** |
| Round II | 45.06±73.79 | 48.88±84.68 | 34.74±55.41 | **0.009** |
| Round III | 48.92±128.31 | 54.30±126.38 | 31.59±383.81 | 0.960 |
| **PM_10_** |  |  |  |  |
| Round I | 160.9±112.89 | 176.48±105.12 | 111.75 ± 86.42 | **<0.001** |
| Round II | 113.72±77.50 | 129.97±72.39 | 88.33±26.59 | **<0.001** |
| Round III | 160.42±164.64 | 160.42±130.56 | 271.81±709.09 | 0.998 |

Mann Whitney test analysis showing the variations of airborne particles present in the breathing zone of participants across the sampling period.

Abbreviations: PM_2.5_- particulate matter of aerodynamic size ≤2.5µm, PM_2.5–10_(coarse) - The different in concentration between PM1_0_ and PM_2.5_. PM_10_ particular matter of aerodynamic size≤10µmNotation: PM_2.5_- particulate matter of aerodynamic size ≤2.5µm, _PM10-2.5_(coarse) - The difference in concentration between PM_10_ and PM_2.5_. PM_10_ particular matter of aerodynamic size≤10µm. Rounds I, II and III corresponds to seasonal changes prevalent in the study areas (i.e. dry, rainy and harmattan) seasons. Bold *p*-values represent significant values (*p* ≤ 0.05).

**Table S2: Associations of selected covariates on the association of PM exposure on HRV indices**

| **Variable** | **RMSSD β[95% CI]** | ***P-*value** | **SDNN β[95% CI]** | ***p-*value** | **LF β[95% CI]** | ***p*-value** | **HF β[95% CI]** | **p-value** | **LF/HF β[95% CI]** | ***p*-value** |
| --- | --- | --- | --- | --- | --- | --- | --- | --- | --- | --- |
| **HR(beats/min)** | -1.43(-3.45,0.59) | 0.17 | -1.06(-2.97,0.85) | 0.28 | -0.21(-0.05,0.01) | 0.16 | -0.02(-0.06,0.01) | 0.21 | 0.024(0.30, 0.08) | 0.39 |
| **Location** | 7.71(2.27,7.81) | 0.01 | 4.82(-5.20,10.56) | 0.77 | 0.57(-0.22,1.36) | 0.16 | 0.69(-0.33,1.70) | 0.18 | 8.87(5.51,12.24) | **0.00** |
| **Job Category** |  |  |  |  |  |  |  |  |  |  |
| Burners | -0.59(-0.96,0.21) | **0.00** | -0.34(-0.61,-0.08) | **0.01** | -1.14(-2.08, -0.19) | **0.02** | -1.26(-2.35,-0.16) | **0.03** | -9.43(12.80,-6.01) | **0.00** |
| Dismantlers | -0.62(-0.94,-0.29) | **0.00** | -0.29(-0.53,0.07) | **0.01** | -0.94(-1.74,-0.13) | **0.02** | -0.99(-1.92,-0.06) | **0.04** | -8.68(11.95,5.41) | **0.00** |
| Sorters | -0.64(-1.28,-0.01) | **0.05** | -0.29(-0.73,0.16) | 0.21 | 0.26(-1.33,1.85) | 0.75 | -0.15(-1.99,1.69) | 0.88 | -7.79(11.95,-3.69) | **0.00** |
| Collectors | -0.98(-1.62,-0.33) | **0.00** | -0.37(-0.82,0.08) | 0.11 | -0.63(-2.24,0.98) | 0.44 | -1.63(-3.49,0.23) | 0.09 | 8.92(5.49,12.35) | **0.00** |
| **Current smokers** | 0.32(0.02,0.62) | **0.03** | 0.18(-0.03, 0.39) | 0.09 | 0.33(-0.41,1.07) | 0.39 | 0.8(-0.01,1.71) | **0.05** | -0.93(-2.45, 0.58) | 0.23 |
| **Age (years)** | 3.62(-0.22,6.02) | **0.02** | 0.01(-0.01,0.01) | 0.97 | -0.01(-0.04,0.04) | **0.03** | -0.01(-0.05,0.05) | 0.96 | -0.05(0.14,0.04) | 0.25 |
| **BMI (overweight)** | -0.16(-0.47,0.16) | **0.02** | -0.11(-0.33,0.12) | 0.34 | -0.64(-1.43,0.15) | 0.06 | -0.27(-1.18,0.65) | 0.57 | 0.027(0.23,0.26) | 0.82 |
| **Cooking Indoor** | -0.01(-0.32,0.31) | 0.97 | -0.04(-0.26,0.18) | 0.72 | -0.13(-0.91,0.66) | 0.75 | 0.18(-0.72,1.09) | 0.69 | 4.08(2.17,10.34) | 0.20 |
| **Hypertension** | 0.13(-1.31,1.57) | 0.86 | -0.04(-1.06,0.98) | 0.94 | -1.32(-4.94,2.30) | 0.48 | -1.86(-6.05,2.33) | 0.38 | 3.64(2.96,10.23) | 0.28 |
| **Diabetes mellitus** | 0.74(-1.22,2.69) | 0.46 | 0.67(-0.71,2.05) | 0.34 | 4.32(-0.57,9.21) | 0.08 | 3.88(-1.78,9.54) | 0.18 | -3.19(11.69,5.31) | 0.46 |
| **Hyperlipidemia** | -0.29(-1.67,1.07) | 0.67 | -0.24(-1.21,0.73) | 0.63 | -2.76(-6.20,0.68) | 0.12 | -1.53(-5.51,2.45) | 0.45 | -1.02(6.03,5.99) | 0.99 |
| **Ambient temp (ºC)** | 3.52(-11.55,18.59) | 0.65 | 5.12(-8.80,19.06) | 0.47 | 0.10(-0.11,0.32) | 0.34 | 0.08(-0.19-0.35) | 0.34 | -0.059(-0.22,0.11) | 0.48 |
| **Relative humidity %** | 0.89(-5.22,7.02) | 0.77 | 1,09(-4.64,6.84) | 0,71 | 0.017(-0.69,0.10) | 0.70 | 0.02(-0.26,0.13) | 0.66 | -0.15(0.55,0.24) | 0.45 |
